# Modulatory effects of R10 fraction of garlic (*Allium sativum* L.) on hormonal levels, T cell polarization, and fertility-related genes in mice model of polycystic ovarian syndrome

**DOI:** 10.1186/s13048-021-00926-6

**Published:** 2022-01-06

**Authors:** Somaye Falahatian, Raheem Haddad, Nafiseh Pakravan

**Affiliations:** 1 Department of Agricultural Biotechnology, Faculty of Agriculture and Natural Resources, Imam Khomeini International University, Qazvin, Iran; 2 Department of Immunology, Medical School, Alborz University of Medical Sciences, Nabowat Blvd, West Bou-Ali St, Karaj, Iran

**Keywords:** PCOS, Cyst, Testosterone, Estradiol, Progesterone, T cell polarization, IFN-γ, IL-4, IL-17, Gpx3, Ptx3, R10 fraction, Garlic

## Abstract

Polycystic ovary syndrome (PCOS) is an inflammatory endocrine-metabolic disorder related to reproductive system characterized by polycystic ovarian morphology, androgen excess, and chronic anovulation. Current treatments haven’t been very successful in PCOS treatment and the problem still remains as a challenge. Therefore, new approaches should be applied to overcome the disease. Previous studies demonstrated immunomodulatory effects of R10 fraction of garlic in the treatment of inflammatory conditions such as cancer. Considering previous studies suggesting immunomodulatory therapy for PCOS, therapeutic effects of R10 fraction was evaluated in a mouse model of PCOS. To do so, PCOS was developed by intramuscular injection of estradiol valerate. Treatment with R10 fraction, isolated from garlic, was performed and the alterations in hormonal levels (estradiol, progesterone, and testosterone), T cell polarization markers (IFN-γ, IL-4, and IL-17), and expression of fertility-related genes (Gpx3 and Ptx3) were evaluated. The results showed that hormonal levels were elevated in PCOS model comparing to normal animals but were markedly modulated after treatment with R10 fraction. Moreover, a severe disturbance in T cell polarization with a significant reduction of fertility-related genes expression were detected in PCOS-induced ovaries. Treatment with R10 fraction also represented modulatory effects on T cell polarization by increasing IL-4 and decreasing IL-17 and IFN-γ levels. Accordingly, fertility-related genes were also modulated following treatment with R10 fraction in PCOS. Our study elucidated that R10 fraction of garlic possess immunomodulatory effects alleviating PCOS symptoms. This approach could be adjusted to give rise the optimum therapeutic results and considered as a candidate therapeutic approach for PCOS.

## Introduction

Polycystic ovary syndrome (PCOS) is one of the most challenging inflammatory disorders related to female reproductive system affecting 5–15% of adult [1, 2]. As a common heterogeneous and reproductive disease, PCOS causes to serious complications including irregularity in menstrual cycles, polycystic ovarian morphology, ovulatory dysfunction, hyperandrogenism, hirsutism, acne, and insulin resistance [1, 3–5]. Normal levels of hormones play a crucial role in normal function of ovaries and regulation of the menstrual cycle. On this basis, persistent hormonal fluctuations lead to the abnormalities such as chronic anovulation, the formation of numerous cysts, and ultimately infertility along with the elevated androgen which is a male hormone causing acne and hirsutism [6, 7]. As a chronic inflammatory disease, PCOS patients have permanently elevated levels of inflammatory markers affecting endothelia cell and endometrium as well as ovaries. On this basis, it has been noted that women with PCOS show an increased risk for type 2 diabetes mellitus, hypertension, obesity, cardiovascular diseases, carcinoma, and psychosexual disorders [2, 8, 9].

Both environmental and genetic factors have been accounted as determinants of the epidemiology and emergence of PCOS. The environmental factors involved in PCOS pathogenesis include life style, diet, environmental toxins like chemical pollutants, and geography and socioeconomic status [10]. Furthermore, genetic risk factors, such as candidate genes, epigenetic, SNPs and ethnicity have shown to play an important role in incidence of PCOS [11]. Recently, the genome-wide association study identified some susceptible *loci* in human genome, containing single nucleotide variants with the strong risk for PCOS in different populations [12]. The multifactorial nature of PCOS has made it difficult to find a potent therapeutic strategy and often multiple treatments are required in parallel to alleviate almost all pathological features of this heterogeneous syndrome [13, 14]. Therapeutic approaches include change of life style & diet, weight loss, exercise, along with pharmacological treatments to modulate the serum levels of their sexual hormones, revert the ovarian functions to normal, improve the metabolic disorders, and modulate inflammatory status related to PCOS [15, 16]. Despite of the striking progressions in the development of potent medicines for PCOS, there is currently no an ideal medical therapy that could treat all PCOS-associated disturbances and the commercially available medicines show various side effects which might in turn cause additional inconvenience for patients. Therefore, new approaches are required to overcome the disease. Since chronic inflammation has emerged as a key contributor to the pathogenesis of PCOS, a great attention has been paid towards immunologic approaches [2, 9, 17].

Garlic or *Allium sativum* [18, 19] is a species in the onion genus, has historically been known as a medicinal plant, and is considered as a valuable plant manifesting blatant therapeutic effects on a range of different diseases [20]. Garlic has widely been prescribed by traditional medicine for long time to treat inflammatory and metabolic disorders including diabetes mellitus, hypertension, cardiovascular diseases and cancers [20, 21]. In addition, the effects of garlic on PCOS have been assessed before [22–24]. On the other hand, previous studies demonstrated immunomodulatory effects of R10 fraction of garlic in the treatment of inflammatory conditions such as cancer [25, 26]. Considering previous studies suggesting immunomodulatory therapy for PCOS [2, 9, 17], this study presented here aimed to evaluate the potential immunomodulatory effects of R10 fraction of garlic in a mouse model of PCOS.

## Material and methods

### Preparation of residue 10 (R10) fraction of garlic

R10 fraction was prepared from Hamedan province garlic (*Allium sativum* 1753 No 2-1912, 14.06.2002) which was previously reported by The Iranian Society of Pharmacognosy [18, 19] as described before [25, 26]. Briefly, the bulbs of garlic were peel off, washed, chopped in small pieces, added into two parts of distilled water, and homogenized in a blender. The resulting homogenate was filter through a Whatman paper under vacuum condition. Then, the obtained extract was centrifuge at 5000 rpm for 30 min, and the supernatant was collected and store at 4 °C. To extract R10, the extract was purified using Amicon ultrafiltration system containing different membranes including 300, 100, 50, 30 and 10 kDa. The fractions were separated, the resulting residual fraction from 10 kDa membrane was collected as fraction R10, and SDS electrophoresis was performed on the final product.

### PCOS induction

Induction of the disease was performed after approval of Imam Khomeini International University authorities under reference No. 8567. The NMRI mice (8 weeks old, 25-30 g weight) were purchase from Pasteur Institute, Tehran, Iran and treated according to the standard ethical protocol related to working with laboratory animals. Sixty female mice were considered and divided into five groups (*n* = 12) including normal control, PCOS-induced group, R10 fraction-treated PCOS group 1, R10 fraction-treated PCOS group 2, and saline-treated PCOS (sham) group. To induce PCOS, the female mice with normal estrous cycle were selected and subjected to vaginal smear assay to determine the steps of estrous cycle [27]. The mice at their estrus phase were received estradiol valerate (40 mg/kg, Sigma Chemical Co., Germany) by a single intramuscular injection and followed up for 8 weeks until PCOS was established.

Estrous cyclicity was monitored daily by analysis of vaginal smears, 2 weeks after injection of estradiol valerate. About 8 weeks after injection, abnormal estrous cycles and persistent vaginal cornification (PVC) were observed that are two main signs of the presence of ovarian cysts and PCOS induction.

### Treatment schedule with R10 fraction

Treatment of PCOS mice with the purified R10 fraction was performed by intraperitoneal injection (20 mg/kg) based on previous studies and unpublished studies of Professor Hassan [25, 26], according to two different strategies including (1) daily treatment during 1 week (R10 Treat 1) and (2) every-other-day treatment for 2 weeks (R10 Treat 2). Three days after the last treatment and at the estrous phase, the mice were sacrificed with ketamine/xylazine and then the blood samples were taken from the myocardial tissue of mice. The serum samples and ovarian tissues were isolated and stored for further evaluations.

### Hormone assessment

For hormonal assay, the serum levels of sexual hormones including testosterone, estrogen and progesterone were evaluated using ELISA kits specific for each hormone (Mybiosurce kits, USA). The assays were run according to manufacturer’s instruction and in triplicate.

### Flow cytometric analysis of T cell polarization markers

The levels of the selected T cell polarization markers including IL-4, IL-17 and IFN-γ were measured using flow cytometry. To do so, the cells were isolated from the ovarian tissues related to untreated and R10-treated PCOS groups by enzymatic isolation. Then, the isolated cells were fixed with 4% paraformaldehyde for 10 min at room temperature and permeabilized with 3% Triton X-100 in PBS for 30 min at room temperature. The samples were incubated with 10% goat serum for 30 min and stained with primary antibodies against IL-17, Il-4, or IFN-γ (1:100 dilution in PBS) for overnight at 4 °C. Next step, the cells were washed once with PBS and incubated with secondary antibody FITC–coupled IgG (1:100 dilution in PBS) for 60 min at 37 °C in the dark. Finally, the cells were washed with PBS, centrifuged at 1500 rpm for 5 min, and the pellet was resuspended in 300 μl PBS to be evaluated using a flow cytometer (BD FACSCalibur™). The data were analyzed using Flowjo 7 software.

### Tissue preparation for hematoxylin and eosin (H&E) staining

The dissected ovarian tissues were fixed in neutral buffered 10% formalin, followed by paraffin embedding and 5 μm-thick sections were prepared on a rotary microtome (Leica, Germany), placed on polylysine-coated slides, and used for the H&E staining. To do H&E staining, based on the routine protocol the sections were rehydrated using differential alcohol gradients for subsequent H&E staining, dehydrated using grade series of alcohol, and immersed in xylene, and mounted in neutral Entellan. Number of primary follicles, preantral follicles, primordial follicles, corpus luteum, and cysts were evaluated in five microscopic fields visualized under a light microscope (Olympus, Japan) by an expert blinded to the experiments and average was considered.

### Quantitative assays

Real-Time PCR was carried out on Gpx3 (Glutathione peroxidase 3) and Ptx3 (Pentraxin 3) genes as two key factors in ovulation and oocyte fertilization. Total RNA was isolated from ovarian tissues according to QIAzol lysis reagent protocol (QIAGEN Inc., Valencia, CA) and the first strand cDNA was synthesized through reverse transcription reaction using cDNA synthesis kit (Thermoscientific, UK). All primers were designed using Gene runner software (Version 6.5.52) and their efficiency and specificity were checked by BLAST tool. The sequences for primers were as forward 5′- TCAGAGCAAGAGAGGCATCC-3′, and reverse 5′-GGTCATCTTCTCACGGTTGG-3′ for beta-actin (Actb), forward 5′-GTGGTGTCTGTATGAAGGAGGG-3′ and reverse 5′-GGGTAGATGGGGGTGTTGAG-3′ for Gpx3, and forward 5′-TAGACGAATGAAGGAGAGA-3′ and reverse 5′- TGTTTAGTTATGTGAGGGTT-3′ for Ptx3. The primers efficiency and specificity as well as fidelity of real-time PCR, and melting curve analysis were controlled as described before [28, 29]. The amplification reactions were run using 2X Real-Time PCR Master Mix (BioFACT™, Korea) and an Applied Biosystems StepOne Real-Time PCR System. To normalize the expression levels of the two genes, Actb was considered as the housekeeping gene. The PCR thermal conditions were set as follows: 95 °C for 15 min as initial denaturation, followed by amplification step for 40 cycles with denaturation at 95 °C for 15 s, annealing at 60 °C for 30 s, and extension at 72 °C for 30 s. A melting curve analysis was also performed at the end of the amplification step. All experiments were carried out in triplicate and the relative expression of genes was analyzed according to the 2^-∆∆Ct^ method.

### Statistical analysis

All experiments were conducted in triplicate and the data were illustrated as the mean ± SEM. Statistical analyses were performed with GraphPad Prism 6. All data were compared using one-way ANOVA test with post hoc Tukey’s test. A *p*-value of 0.05 was considered as significant difference.

## Results

### Modulation of hormonal perturbations in PCOS model by R10 fraction

**Fig. 1:**
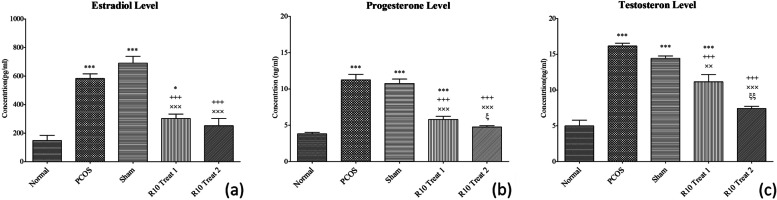
R10 fraction effect on serum levels of **a** estradiol, **b** progesterone, and **c** testosterone were determined subsequent 3-day in normal, PCOS, sham, R10 Treat 1, and R10 Treat 2 groups (*n* = 12). Data are expressed as the mean ± SEM. Significant difference vs. the normal (*), vs. the PCOS group (+), vs. the sham group (×), and vs. the R10 Treat 1 group (ξ)

Given the importance of hormonal modulation following PCOS treatment [6, 7], levels of testosterone, estrogen, and progesterone hormones were initially evaluated. As shown in Fig. 1, the levels of progesterone, estradiol, and testosterone were increased in PCOS model compared to normal group (*p* < 0.001). However, both of treatments regimens (one-week: R10 Treat 1 or two-week: R10 Treat 2) using R10 fraction led to significant modulation in the serum levels of all three hormones of interest in PCOS-treated animals (*p* < 0.001). R10 treat 2 showed more significant impact than R10 Treat 1 on the three hormones (*p* < 0.01).

**Fig. 2:**
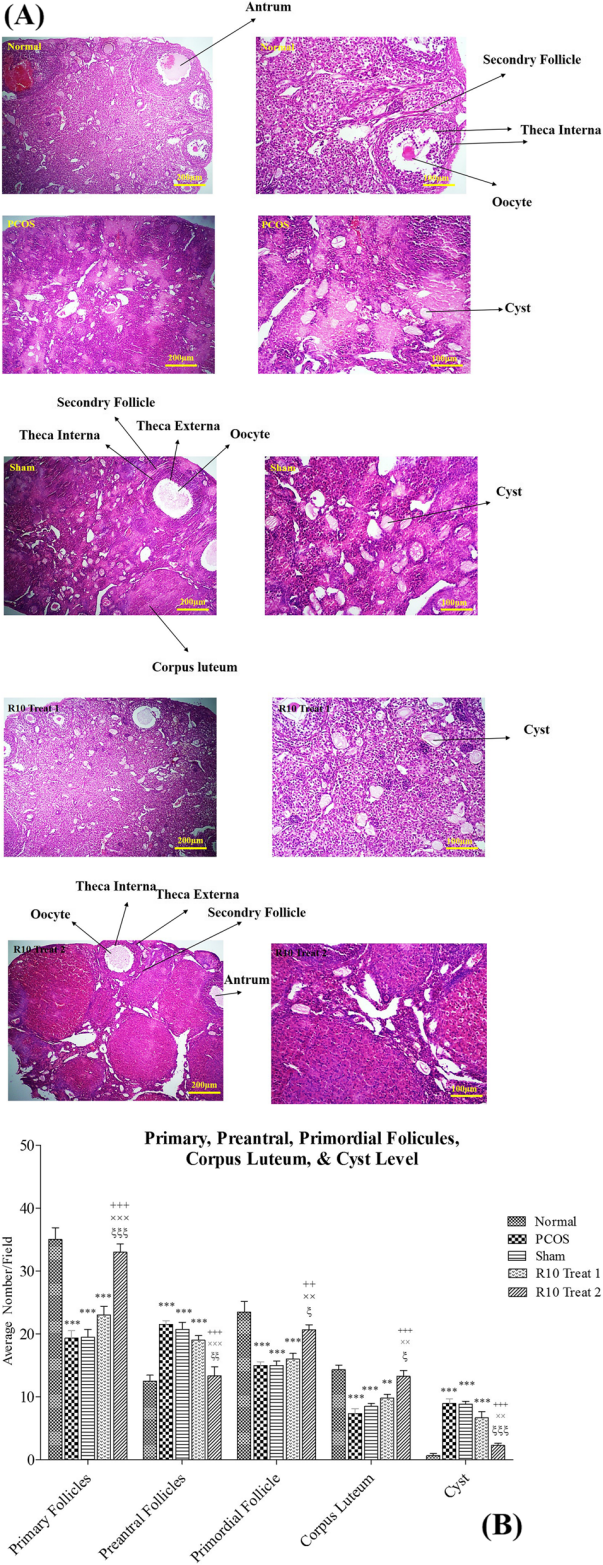
Ovarian tissues isolated from normal, PCOS, sham, R10 Treat 1, and R10 Treat 2 groups were stained with H&E (**A**) and average number of follicles and cyst (**B**) were examined. Data are expressed as the mean ± SEM. Significant difference vs. the normal (*), vs. the PCOS group (+), vs. the sham group (×), and vs. the R10 Treat 1 group (ξ). Scale bar unit: μm

The ovary tissues were next examined for the presence of cyst. H&E staining (Fig. 2A, B). The results indicated that in PCOS-induced ovaries, the number of primary and primordial follicles was significantly decreased and consequently the number of preantral follicles was considerably increased comparing with the normal ovaries. Notably, treatment with R10 fraction in R10 Treat group could prevent the abnormal maturation of follicles and, as a result, preventing depletion of the ovarian follicles reservoir. As expected, the number of corpus luteum was higher in normal group indicating normal ovulation. Conversely, the PCOS ovarian samples showed a significant decrease in the number of corpus luteum. Interestingly, the number of corpus luteum was significantly increased in R10 Treat 2 group implying significant improvement in the normal ovulation process and better therapeutic results than R10 Treat 1 group (Fig. 2B). Consistently, examination of H&E-stained tissue revealed that the presence of ovarian cysts was markedly increased in PCOS-induced mice comparing to the normal group. Again, treatment with R10 fraction in R10 Treat 2 group could effectively reduce the number of cysts, while the number did not significantly change in the R10 Treat 1 group.

### Immunomodulatory effects of R10 fraction on T cell polarization in polycystic ovaries

**Fig. 3:**
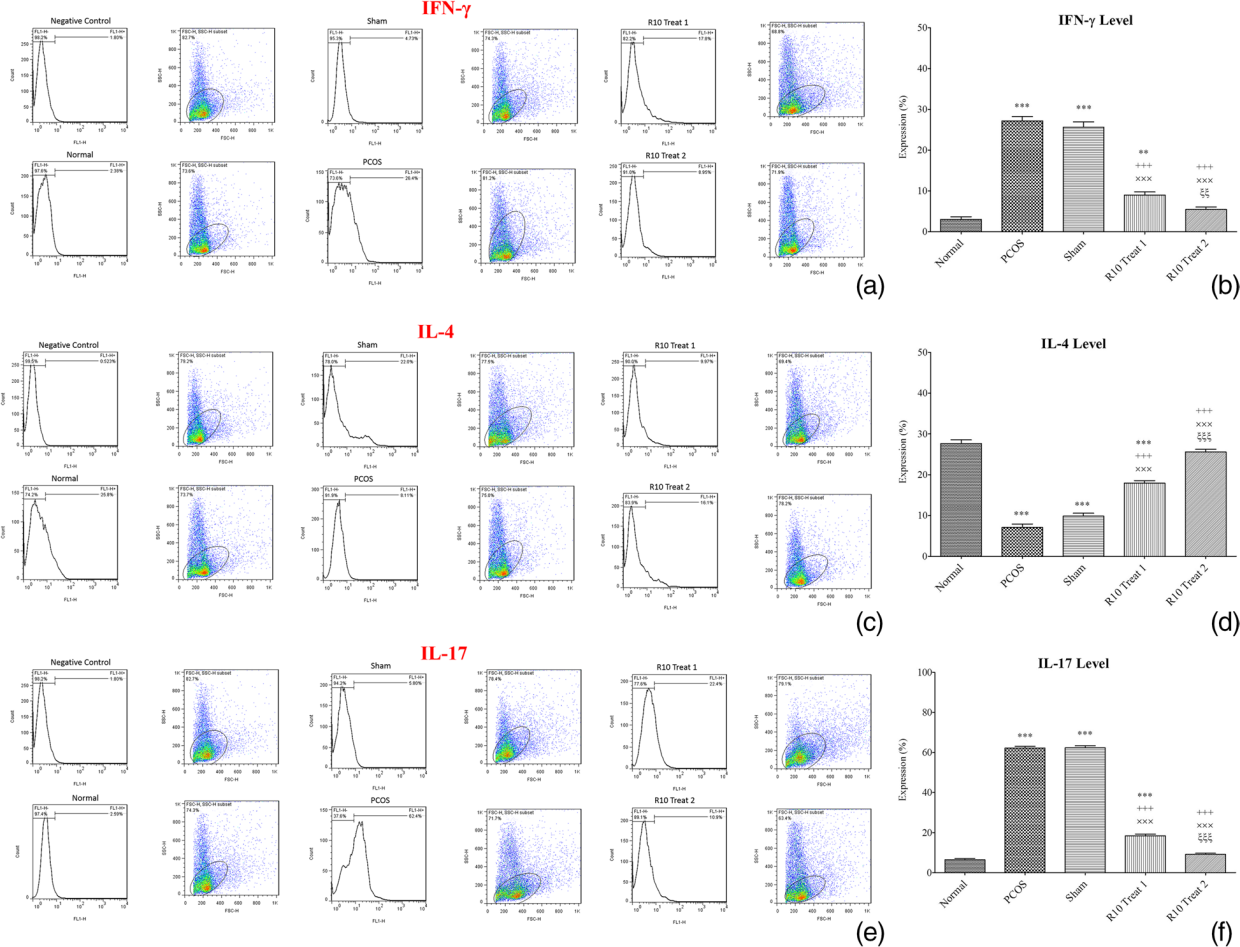
The alterations of IFN-γ, IL-4, and IL-17 levels in ovary tissue of normal, PCOS, and R10 fraction-treated groups (*n* = 12). Representative flow cytometry plot of the immunostained cells (**a**, **c**, **e**) and the percentage of IFN-γ, IL-4, and IL-17 expression (**b**, **d**, **f**). IFN-γ, IL-4, and IL-17 were detected by immunostaining of ovarian cells using the corresponding antibody for each group. Data are presented as mean ± SEM. Significant difference vs. the normal (*), vs. the PCOS group (+), vs. the sham group (×), and vs. the R10 Treat 1 group (ξ)

T cell polarization has been suggested to have important role in immunophathogenesis of PCOS [22–26]. To evaluate if R10 fraction has immunomodulatory effects on mice model of PCOS, IFN-γ, IL-4, and IL-17 were measured as representatives of Th1, Th2, and Th17, respectively [28, 29]. As illustrated in Fig. 3, IL-17 and IFN-γ levels were enhanced in ovarian tissues of the mice affected by PCOS (*p* < 0.001), indicating the involvement of T cell polarization in PCOS. On the contrary, IL-4 level was significantly reduced in PCOS status (*p* < 0.001). Influenced by R10 fraction treatments, IL-17 and IFN- γ exhibited a significant decrease and IL-4 was elevated in R10-treated PCOS groups (*p* < 0.001). Treatment 2 had a marked immunomodulatory effects on T cell polarization comparing with treatment 1 (*p* < 0.01).

### Regulatory impact of R10 fraction on specific genes involved in ovulation and oocyte fertilization

**Fig. 4:**
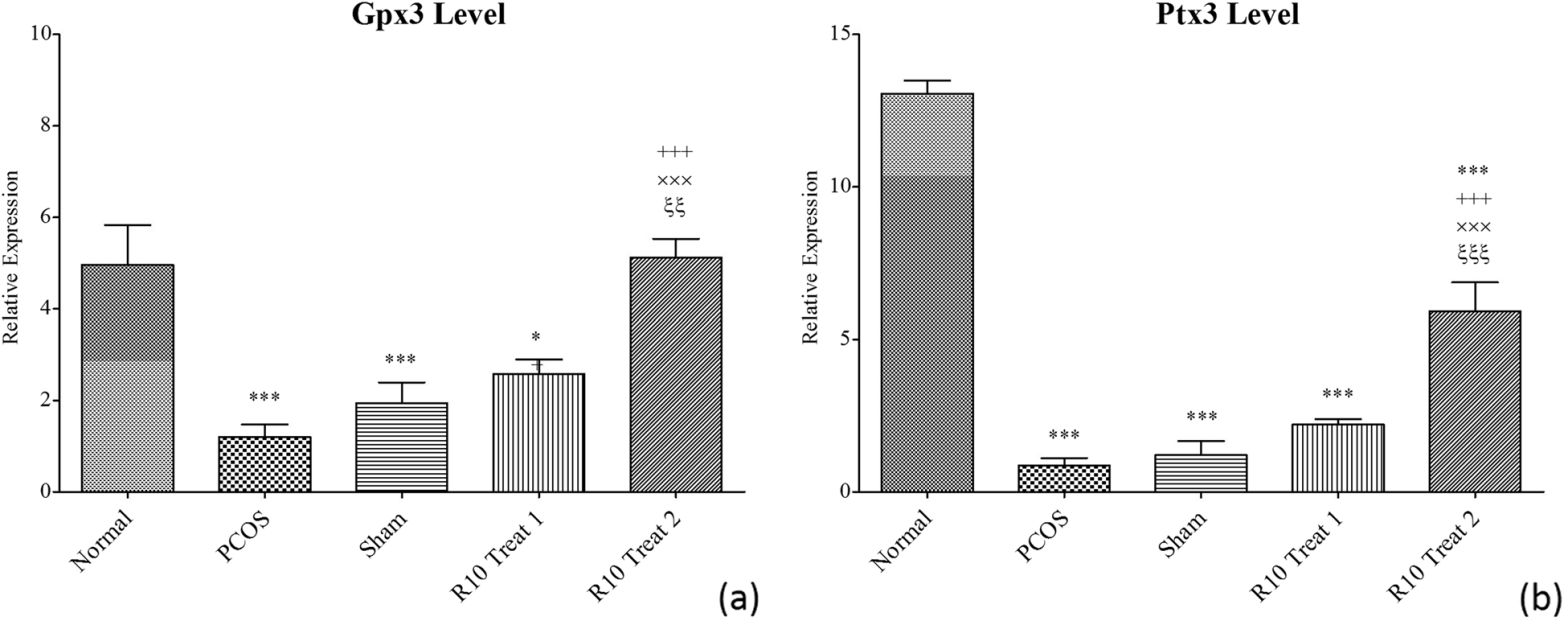
Comparison of mRNA expression of Gpx3 (**a**) and Ptx3 (**b**) in the ovary of normal, PCOS, sham, R10 Treat 1, and R10 Treat 2 groups (*n* = 12). The last two groups of animals were treated via intraperitoneal route 8 weeks after the disease induction. The animals were euthanized 3 days after the last treatment. The quantification of each gene was normalized against the reference gene as described in the text. Data are presented as mean ± SEM. Significant difference vs. the normal (*), vs. the PCOS group (+), vs. the sham group (×), and vs. the R10 Treat 1 group (ξ)

Results from quantitative PCR showed that the expression levels of both Gpx3 and Ptx3 genes, as the two critical factors in normal ovulation and fertility, were drastically decreased in the PCOS mice compared to the normal group (Fig. 4, *p* < 0.001). Treatments with R10 fraction manifested an impressive effect on upregulation of Gpx3 and Ptx3 genes. It was observed that the two-week treatment strategy (R10 Treat 2) led to a significant increase in Gpx3 gene expression up to normal level comparing to PCOS and sham groups. However, in case of Ptx3 gene, the expression level following R10 Treat 2 was significantly lower than normal group (*p* < 0.001) and higher that PCOS and sham groups (*p* < 0.001). No statistically significant upregulation of Gpx3 or Ptx3 was observed following one-week treatment (R10 Treat 1) as compared to PCOS and sham groups. In general, it seems that R10 Treat 2 was more effective than R10 Treat 1 in modulating the expression of the two genes.

## Discussion

PCOS is one of the difficult treating diseases. While current therapeutic approaches do not include a single gold-standard agent, it mainly depends on the clinical features of each patient aiming to improve fertility, regulate menstrual disturbances, mitigate the symptoms of hyperandrogenism, obesity treatment, and manage the metabolic disorder [30]. Amongst them, metformin and supplementation with garlic as herbal medicine along with vitamin D & omega-3 are the most common medications currently used in PCOS treatment. Metformin reduces insulin resistance, improves the lipid profile, decreases blood pressure, and affects some of sex hormone level [21, 23, 24, 31]. However, clinical sign-based therapies may affect other aspects of the disease. For example, metformin modulates metabolic & hormonal aspect of the disease, but its effects on T cell polarization and balance of IL-4, IL-17, and IFN-γ is still a matter of controversy [32–35]. Considering the role of immune system in maintaining homeostasis of the body [36], it is important to find an immunotherapy-based treatment for PCOS. Apart from pharmacological therapies administered for PCOS treatment, herbal plants recommended by traditional medicine, such as *Tribulus terrestris* and licorice, have been addressed for their beneficial therapeutic effects on PCOS [37–40]. However, no efficient fraction from the candidate herbs has been determined and isolated.

It has become evident that PCOS is not an organ-specific pathological condition affecting only the ovaries, but it is a multisystem disease with a close correlation to other systemic metabolic disorders. It seems that systemic inflammation causes apoptosis in the ovarian granulosa and theca cells leading to disturbance of ovulation [41]. Consistently, none of the current clinical symptoms-targeted therapies have achieved desirable systemic outcome. On this basis, a recent goal of the nowadays research is to understand the molecular mechanisms and role of the chronic inflammation involved in the pathogenesis of PCOS. This is an important issue because it also helps devise novel immunomodulatory approaches as the future options of treatment for patients with PCOS. Immunosuppression and antioxidant agents have been previously addressed [31]. However, this area is still in its infancy and more research and clinical trials are required to reveal the optimum medication.

In traditional medicine, garlic is well-known due to its great medical properties and utilized from a long time ago for the treatment of a variety of different diseases [42]. On the other hand, PCOS is known as a risk factor for tumor development [43] and R10 fraction of garlic has shown immunomodulatory effects on tumor [25, 26, 44]. These two points prompted us to evaluate therapeutic effects of R10 fraction on PCOS. To do so, immunomodulatory effect of R10 fraction on the modulation of hormonal levels and T cell polarization markers were evaluated in a mice model of PCOS. Moreover, we evaluated potential regulatory effects of R10 fraction on the alterations of Gpx3 and Ptx3 gene expression, as two key genes in oocyte development and fertilization. Our results demonstrated that the serum testosterone, estradiol, and progesterone levels were considerably elevated in the PCOS comparing to the normal animals, implying on abnormalities in the metabolism of estrogen and androgen hormones in PCOS models consistent with previous reports [45–47]. R10 fraction treatments could considerably exert efficient therapeutic effects on the modulation of hormonal levels in proportion to PCOS. Partial modulatory effects of garlic extract on sexual hormones was previously shown in male rats under treatment with cyclophosphamide [48] but not in female animal of PCOS model [49]. This study introduces the specific fraction of garlic to obtain optimum therapeutic effects. In addition, a research has shown that garlic affects the activity of key enzymes in the conversion of cholesterol to testosterone [50]. Alternatively, garlic has also been shown to partially correct imbalances in estrogen metabolism and stimulate the secretion of gonadotropins and ovarian hormones in monosodium glutamate-induced fibroid in female Wistar rats [51]. Consistent with sex hormone modulation and histologic evaluation using H&E, our results revealed that the R10 fraction could also exert its modulatory effects on T cell polarization. IL-17 and IFN-γ as representative of Th17 and Th1, respectively, were significantly upregulated in PCOS models of mice. Inversely, IL-4, as representative of Th2 response was dropped below normal level, suggesting polarization of T cell response in polycystic ovaries. This is consistent with the previous study performed on serum or follicular fluid from women affected by PCOS [52–56]. T cell polarization markers have even been suggested as a predictor of infertility in women or female mice with PCOS [57]. Such a T cell polarization pattern is followed by secondary morbidities of PCOS including insulin resistance, hypertension, and chronic inflammation. Foroozanfard et al. reported that the blood pressure is directly correlated with the serum IL-17 level in women with PCOS. They found that the IL-17 level in patients with high blood pressure was abnormally higher than normal women [58]. Our flow cytometry data showed that R10 fraction applied led to a significant modulation of T cell polarization represented by decreasing IL-17 & IFN-γ and enhancing IL-4 level. Consistently, Kaschula et al. also showed that garlic could somehow contribute to the reduction of chronic inflammation as an anti-inflammatory supplement that might partially reduce the risk of certain types of cancers [59]. Another study conducted in 2011 demonstrated that garlic was able to partially reduce liver inflammation caused by changes in the levels of cytokines IL-6 and IFN-γ in mice with a protozoan parasite [60]. To treat inflammatory diseases, such as PCOS, the important point is that to modulate (not suppress) the immune response in a way to deviate the inflammatory process into limiting phase [61]. Previous studies demonstrated that R10 fraction modulates inflammatory process by affecting innate and adaptive immune system [25, 26, 62–65]. As shown in this study, R10 fraction modulated T cell response which was concomitant with reduced cyst level suggesting entrance of the inflammatory process into limiting phase. However, repeated treatment courses may require as environmental factors and life style are also involved in PCOS [66]. More experiments may help optimize protocol and/or dose of treatment to get better results at a shorter time.

In line with the modulation of T cell polarization and inflammatory status the two master regulatory genes including oocyte-secreted factors including Gpx3 and Ptx3 were also modulated upon treatment with R10 fraction comparing with the PCOS mice which had severely downregulated Gpx3 and Ptx3 expression. It has previously been demonstrated that these two important genes are associated with oocyte maturation and embryo quality and drastically reduced in women with PCOS [67]. Previous reports asserted that deletion or reduction of Ptx3 and Gpx3 expression levels in mice results in the failure of oocyte fertilization and infertility [68]. The two genes are also affected in human cases of PCOS [69, 70]. Our quantitative data indicated that two-week treatment with R10 fraction (R10 Treat 2) impressively upregulated expression of Gpx3 and Ptx3 genes in comparison to PCOS group, indicating that R10 fraction was also effective in gene regulation of polycystic ovaries. Regarding the effect of using garlic on Gpx3 gene expression, a study found that using garlic in the diet of fish exposed to aflatoxin toxin could partially increase the expression of this gene in their tissues [71].

A notable issue in this study was the effect of different treatment regimen with R10 fraction. The results of this study demonstrated that treatment with R10 fraction every other day for 2 weeks (R10 Treat 2) had more beneficial effects that every day treatment for 1 week (R10 Treat 1) which was selected based on the previous studies and unpublished studies of Professor Hassan [25, 26]. There are studies showing that therapeutic effects are influenced by dosing and/or timing of medication administration. Current regulation of Food and Drug Administration (FDA) generally only require a medication to be markedly better comparing to placebo [72]. Suboptimal exposure can then lead to poor efficacy as demonstrated for R10 fraction in this study. A challenge for new therapeutic approaches, the right dosing and/or dosing can be adjusted by therapeutic monitoring and evaluation of appropriate biomarkers in preclinical experiments.

## Conclusion

Currently, there is no definite cure for PCOS and current therapies include metabolic or hormonal approach to manage individual’s concerns such as infertility, obesity, acne, or hirsutism by affecting metabolic or hormonal aspects. Such clinical sign-based therapies, e.g. metabolic, may affect other aspects of the disease. For example, metformin modulates metabolic aspect of the disease, but its effects on T cell polarization and balance of IL-4, IL-17, and IFN-γ is still a matter of controversy. This study proposes R10 fraction as the effective fraction of garlic suggesting it as a potent candidate therapeutic agent for PCOS. R10 fraction is able to achieve a balance in hormonal, immunologic, and genetic aspect of PCOS in concert.

## Data Availability

The data that support the findings of this study are available from the corresponding author, Dr. Nafiseh Pakravan, upon reasonable request.

## Abbreviations

PCOS: Polycystic ovary
IFN-γ: Interferon-γ
IL-4: Interlukin-4
IL-17: Interlukine-17
Th1: T-helper1
Th2: T-helper2
Th17: T-helper17
Gpx3: Glutathione peroxidase 3
Ptx3: Pentraxin 3
